# Rapid Killing and Biofilm Inhibition of Multidrug-Resistant *Acinetobacter baumannii* Strains and Other Microbes by Iodoindoles

**DOI:** 10.3390/biom10081186

**Published:** 2020-08-14

**Authors:** Chaitany Jayprakash Raorane, Jin-Hyung Lee, Jintae Lee

**Affiliations:** School of Chemical Engineering, Yeungnam University, Gyeongsan 38541, Korea; chaitanyaraorane22@ynu.ac.kr (C.J.R.); jinhlee@ynu.ac.kr (J.-H.L.)

**Keywords:** *Acinetobacter baumannii*, antibiotics, biofilm, 5-iodoindole, membrane damage

## Abstract

Multi-drug resistant *Acinetobacter baumannii* is well-known for its rapid acclimatization in hospital environments. The ability of the bacterium to endure desiccation and starvation on dry surfaces for up to a month results in outbreaks of health care-associated infections. Previously, indole and its derivatives were shown to inhibit other persistent bacteria. We found that among 16 halogenated indoles, 5-iodoindole swiftly inhibited *A. baumannii* growth, constrained biofilm formation and motility, and killed the bacterium as effectively as commercial antibiotics such as ciprofloxacin, colistin, and gentamicin. 5-Iodoindole treatment was found to induce reactive oxygen species, resulting in loss of plasma membrane integrity and cell shrinkage. In addition, 5-iodoindole rapidly killed three *Escherichia coli* strains, *Staphylococcus aureus*, and the fungus *Candida albicans*, but did not inhibit the growth of *Pseudomonas aeruginosa*. This study indicates the mechanism responsible for the activities of 5-iodoindole warrants additional study to further characterize its bactericidal effects on antibiotic-resistant *A. baumannii* and other microbes.

## 1. Introduction

*Acinetobacter baumannii* is a notorious bacterium and is recognized as one of the top seven pathogens in terms of the challenge to our healthcare-delivery systems [1,2]. *A. baumannii* is a Gram-negative coccobacillus and a ubiquitous opportunistic pathogen that causes nosocomial infections, which include hospital-acquired pneumonia and bloodstream, urinary tract, skin, and soft tissue infections [3,4,5,6]. Due to its multidrug resistance (MDR), *A. baumannii* thrives in nosocomial environments and prevails in critically ill-patients hospitalized in intensive care units [7,8]. Prevalence of resistance to multiple antibiotics is due to the inherent ability of *A. baumannii* to endure desiccation, disinfectants, and antibiotics [9]. In addition, *A. baumannii* has developed a strategy to survive in dry conditions by forming biofilms, which makes the bacterium extremely difficult to eliminate [10].

Indole and its aromatic heterocyclic structure are widely utilized as synthetic starting points by the pharmaceutical industry [11]. Indole acts as an interspecies and interkingdom signaling molecule and controls diverse bacterial functions [12,13]. Indole and its derivatives, such as 7-hydroxyindole [14], 3-indoleacetonitrile [15,16], 7-fluroindole [17], 2-aminobenzimidazoles [18], 7-benzyloxyindole [19,20] have been reported to attenuate the virulence of and biofilm formation by enterohemorrhagic *E. coli* O157:H7, *Pseudomonas aeruginosa*, *Staphylococcus aureus*, *Candida albicans*, *Paenibacillus alvei*, and *Agrobacterium tumefaciens* [21].

Among a series of halogenated indoles, 5-iodoindole eradicated *E. coli* and *S. aureus* persister cells and biofilms via an unknown mechanism [22] and killed the bacterivorous nematode *Caenorhabditis elegans* [23], pinewood nematode *Bursaphelenchus xylophilus* [24,25], and root-knot nematode *Meloidogyne incognita* [26], via methuosis [27]. Furthermore, 5-iodoindole has been shown to be nontoxic to plants and animals [25,26]. However, the mechanism responsible for the effects of 5-iodoindole in bacteria has not been determined and its efficacy has not been studied against MDR *A*. *baumannii* strains.

In the present study, 24 indole derivatives, including 16 halogenated indoles, were initially screened for antibacterial and antibiofilm activities against *A. baumannii*. Biofilm dispersal, pellicle formation, motility assays, microscopic analyses of biofilm structure and membrane integrity, and reactive oxygen species assays were performed on *A. baumannii*. Three *E. coli* strains, *P. aeruginosa*, Gram-positive *S. aureus*, and the fungus *C. albicans*, were tested for rapid killing activity and microscopic morphological examinations.

## 2. Materials and Methods

### 2.1. Ethics Statement

This study did not involve any human or animal participants.

### 2.2. Bacterial Strains and Chemicals

Nine *A. baumannii* strains, including six MDR clinical isolates, were used in the present study. Media (Luria-Bertani (LB), tryptic soy broth (TSB), and potato dextrose broth (PDB)) were purchased from Becton Dickinson (Franklin Lakes, NJ, USA). The nine *A. baumannii* strains (Table S1) used were routinely maintained and grown in Tryptic soy broth (TSB) broth at 37 °C under shaking conditions. Two *A. baumannii* strains were procured from the American Type Culture Collection (ATCC), *A. baumannii* ATCC 17978 and ATCC BAA-1709 and the seven clinical *A. baumannii* isolates (A 550, A 578, A 553, A 556, A 580, A 571, A 564) were isolated from burns patients at the National Rehabilitation Institute of Mexico. *A. baumannii* ATCC 17978 was used as a reference strain [28]. Experiments were conducted at 37 °C in LB medium for *E. coli* BW25113, *E. coli* O157:H7 EDL933, *E. coli* O6:H1 CFT073, methicillin-sensitive *S. aureus* 6538 (MSSA), and *P. aeruginosa*. *C. albicans* DAY 185 was cultured in PDB. Chemicals and the 24 indole derivatives viz. 4-bromoindole, 5-bromoindole, 6- bromoindole, 7- bromoindole, 4-chloroindole, 5-chloroindole, 6-chloroindole, 7-chloroindole, 5,6-difluroisatin, 2,3-dimethyl indole, 4-fluroindole, 5-fluroindole, 6-fluroindole, 7-fluroindole, indole, 4-iodoindole, 5-iodoindole, 6-iodoindole, 7-iodoindole, isatin, 3-methyl indole, 7-nitroindole, 5-nitroisatin, 5-(trifluromethoxy) indoline-2,3-dione were purchased from Sigma-Aldrich (St. Louis, MO, USA) and Combi-Blocks, Inc. (San Diego, CA, USA) and were dissolved in dimethyl sulfoxide (DMSO), which did not exceed 0.1% (*v*/*v*) in any experiment. Ciprofloxacin, gentamicin, colistin, and crystal violet were purchased from Sigma-Aldrich Co. (St. Louis, MO, USA). Ethanol (95%) was purchased from Duksan Pure Chemicals (Daegu, South Korea), povidone iodine from Gumi pharmaceuticals (Gumi, South Korea) and sodium iodide, potassium iodide, and dimethyl sulfoxide (DMSO) from Duksan Pure Chemicals (Daegu, South Korea). DMSO was also used as a negative control in all experiments at a concentration in media of ≤ 0.1% (*v*/*v*): it did not affect cell growth or antibiofilm activity. Minimum inhibitory concentrations (MICs) of indole derivatives were determined (Table S2) according to the Clinical Laboratory Standards Institute (CLSI) guidelines for bacteria. MIC was defined as the lowest concentration that inhibited cell growth [29].The MICs of indole derivatives were determined using a microdilution method in 96-well polystyrene plates (SPL Life Sciences). Overnight cultures of *A. baumannii* were treated with indole or indole derivatives at various concentrations (0–250 µg/mL) and incubated at 37 °C for 24 h. Briefly, freshly grown cells were diluted in cation-adjusted Mueller Hinton Broth for the optimum size of inoculum (10^5^ cells) for MICs. Experiments were performed using at least three independent cultures.

### 2.3. Crystal Violet Biofilm Assay

The effect of indole derivatives on *A. baumannii* static biofilm formation was assayed in 96-well polystyrene plates as previously reported [30,31]. In brief, cells in TSB (total volume 300 µL) at an initial turbidity of 0.05 at 600 nm (OD_600_) were cultured with or without compounds for 24 h without shaking at 37 °C. The same amount of TSB was added to peripheral wells in 96-well plates to avoid edge effects. To quantify total biofilm formation, biofilms in 96-well plates were stained with 0.1% crystal violet for 20 min, dissolved in 95% ethanol, and then absorbances were measured at 570 nm (OD_570_) using a Multiskan EX microplate reader (Thermo Fisher Scientific, Waltham, MA, USA). Results are the averages of measurements taken from at least six replicate wells.

### 2.4. Biofilm Dispersal Assay

A modified biofilm dispersal assay was used to investigate the disruptive effects and bactericidal action of 5-iodoindole on preformed biofilms of *A. baumannii*, as described previously [32]. Briefly, planktonic cells were removed and fresh TSB medium containing different concentrations of 5-iodoindole (0–250 µg/mL) were added to the wells of 96-well polystyrene plates containing *A. baumannii* biofilms, which had been developed for 12 h at 37 °C in TSB medium, and then incubated for 12 h at 37 °C. Crystal violet biofilm assays were performed as described above.

### 2.5. Pellicle Formation and Motility Assays

Pellicle formation assay were performed as previously described [33]. In brief, overnight bacterial cultures were diluted 1:100 in 5 mL of LB broth and grown in glass tubes for 72 h at 25 °C in the dark without agitation [34]. Amounts of pellicle material were assessed by adding 1 mL of ethanol to tubes underneath pellicle material, removing floating pellicles, and suspending them in phosphate buffered saline (PBS). OD_600_ values were measured using a spectrophotometer (Optizen 2120 UV, Mecasys, South Korea). Experiments were conducted in triplicate on three different days. Motility agar containing 0.4% agarose, 1% tryptone, and 0.5% yeast extract was used to assess surface motilities at different 5-iodoindole concentrations [35]. 5-Iodoindole at 10 and 25 µg/mL was added to motility agar, and DMSO (0.1%) was used as a negative control. Overnight grown ~0.2 µl cultures of *A. baumannii* ATCC 17978 were placed on motility plates using a sterile pipette tip. Sizes of halos produced by cells traveling across agar plates were measured after incubation for 24 h at 37 °C. Each experiment was performed using at least three independent cultures.

### 2.6. Biofilm Examinations by Confocal Laser Scanning Microscopy (CLSM)

*A. baumannii* biofilms were formed in TSB on glass-bottom dishes (SPL Life Sciences, Pocheon, Korea) with or without 5-iodoindole (0, 25, or 50 µg/mL) without shaking for 24 h. Variations in biofilm formation in the presence of 5-iodoindole were assessed by CLSM (Nikon Eclipse Ti, Tokyo, Japan) [30]. Cells were stained with carboxyfluorescein diacetate succinimidyl acetate (Invitrogen, Molecular Probes, Eugene, OR, USA), and samples were visualized using a 20× objective and an Ar laser (excitation wavelength 488 nm, emission wavelength 500 to 550 nm). For each experiment, at least 10 random positions in each of three independent cultures were chosen for microscopic analysis. Spatial characteristics were quantified using COMSTAT biofilm program (http://www.comstat.dk/) by analyzing at least four random positions in three independent cultures. To measure biofilm formation, color confocal images (20 image stacks) were converted to gray scale using ImageJ program (https://imagej.nih.gov/ij/). COMSTAT biofilm software was used to determine biomasses (µm^3^ per µm^2^), mean thicknesses (µm), and substratum coverages (%) [36].

### 2.7. Time-Kill Kinetics Assays

To determine the killing efficacy of 5-iodoindole, time-kill experiments were performed as previously described [5,37]. Briefly, overnight cultures of *A. baumannii* ATCC 17978 strain at a dilution of 1:100 inoculated in TSB were used for assays. Samples were treated with (0, 20, 50, 100 or 200 µg/mL) of 5-iodoindole or ciprofloxacin, gentamicin, and colistin and incubated for 1 h at 37 °C with shaking (250 rpm). At the indicated time points, aliquots of treated cells were harvested, diluted as required, and plated onto TSA agar plates. After overnight incubation at 37 °C, colony forming units (CFUs) were enumerated. Rapid killing activity by the most active compound 5-iodoindole was also investigated against various other microorganisms, that is, one Gram-positive bacteria, 4 Gram-negative bacteria, and *C. albicans*. Cultures were inoculated and treated with 0 or 486 µg/mL (2 mM) of 5-iodoindole in their respective culture media. At the indicated time points, aliquots of treated cells were plated, and CFUs were enumerated. Experiments were performed using at least two independent cultures.

### 2.8. Scanning Electron Microscopy (SEM) of Cell Membrane Integrity

Cell morphology of *A. baumannii* was investigated by SEM as previously described [30]. Cells were grown in 250 mL glass conical flasks containing TSB as medium for 1 h in the presence of 5-iodoindole and/or colistin at 125 and 200 µg/mL and then fixed on nylon membrane filter pieces (0.5 cm × 0.5 cm) using a microfiltration assembly according to a reported method [38]. Cells were fixed with 2.5% glutaraldehyde/2% formaldehyde for 24 h, post-fixed in PBS and osmium tetroxide, and dehydrated using an ethanol series (50, 70, 80, 90, 95, and 100%) and isoamyl acetate. After critical-point drying, cells on filters were sputter-coated with palladium/gold and observed under an S-4100 scanning electron microscope (Hitachi, Tokyo, Japan) at magnifications ranging from x 1000 to 10,000 using an accelerating voltage of 15 kV.

### 2.9. Reactive Oxygen Species (ROS) Assay

ROS generation in *A. baumannii* was assayed as described previously [39]. Briefly, overnight grown cells of *A. baumannii* ATCC 17978 in TSB were harvested, washed and re-suspended at 10^6^ CFU/mL in PBS. The resuspended culture was treated with or without 5-iodoindole (20, 50, 100, or 200 µg/mL), or H_2_O_2_ (10, 20, or 50 µg/mL; positive control) in 14 mL sterile polypropylene tubes for 1 h at 37 °C and 250 rpm. 2′, 7′-Dichlorofluorescein diacetate (5 µM; Sigma-Aldrich, USA) was then added to cell suspensions and incubated in the dark for 30 min at 37 °C. Fluorescence was measured using a multimode microplate reader JASCO-F-2700 (Hitachi, Tokyo, Japan) equipped with a xenon arc lamp. Excitation and emission slits were fixed at 5 nm, respectively, excitation wavelength was set at 506 nm, and emission intensities were recorded at 524 nm. Fluorescence intensities (FI)/OD_600_ were normalized with respect to growth. Untreated cells were processed similarly and used as controls. UV–vis spectra of samples were obtained using a 3220 UV spectrometer (Optizen, Daejeon, Korea) using quartz cuvettes (1 cm path length) at a wavelength uncertainty of ±2 nm from 200 to 800 nm. Results are representative of three independent experiments.

### 2.10. Analyses of Release of Reducing Sugars and Proteins

The effects of 5-iodoindole on membrane release of reducing sugars and proteins released by treated cells was assessed. Briefly, 100 mL LB broth culture was treated with 5-iodoindole (200 µg/mL), incubated for 3 h at 37 °C with shaking (250 rpm), and centrifuged at 10,000 g for 30 min at 4 °C. Supernatants were stored at −20 °C, and reducing sugar levels were estimated using a dinitrosalicylic acid assay [40]; absorbances were recorded at 540 nm. Protein levels were determined using the Bradford method and absorbances were recorded at 595 nm [41].

### 2.11. Statistical Analysis

Replication numbers for assays are provided above and results are expressed as means ± standard deviations. The statistical analysis was performed by one-way ANOVA followed by Dunnett’s test using SPSS version 23 (SPSS Inc., Chicago, IL, United States). Alternatively, Kruskal–Wallis and Mann–Whitney tests were performed. *p* values of <0.05 were regarded significant and asterisks are used to indicate significant differences between treated and untreated samples.

## 3. Results

### 3.1. Antibiofilm and Antimicrobial Activities of Halogenated Indoles against A. baumannii

**Figure 1:**
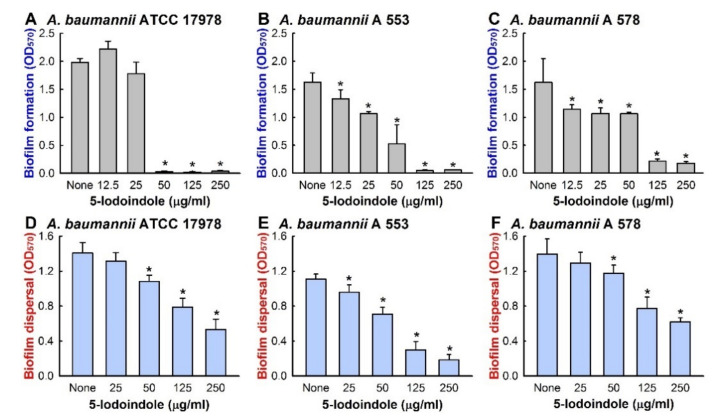
Biofilm inhibition and dispersal of *A. baumannii* by 5-iodoindole. Biofilm inhibitory effects of 5-iodoindole against *A. baumannii* strains (**A**) American Type Culture Collection (ATCC) 17978; (**B**) A 553 and (**C**) A 578 after incubation for 24 h without shaking at 37 °C. Biofilm dispersing effects of 5-iodoindole against *A. baumannii* strains (**D**) ATCC 17978; (**E**) A 553 and (**F**) A 578. Error bars represent standard deviation. * *p* < 0.05 versus untreated controls.

To identify new antibiofilm agents against *A. baumannii*, 24 indole derivatives, including 16 halogenated indoles, were initially screened at a concentration of 50 µg/mL to minimize antimicrobial effects. Of these indoles, 4-bromoindole, 4-chloroindole, 4-iodoindole, 6-iodoindole, and 5-iodoindole showed a significant drop in biofilm mass as measured by crystal violet and the percentage reduction were 62%, 75%, 60%, 94%, and 96%, respectively (Table S3). Of the two most effective indoles, 5-iodoindole inhibited cell growth more than 6-iodoindole, and thus, it was the focus of subsequent studies conducted using the reference *A. baumannii* ATCC 17978 strain. Both 5- and 6-iodoindole dose-dependently inhibited biofilm formation by *A. baumannii* 17978 strain (Figure 1A and Figure S2). The minimum inhibitory concentrations (MICs) of the 16 halogenated indoles against ATCC 17978 fell in the range 50 to 250 µg/mL and the MICs of 5- and 6-iodoindole were lowest at 50 µg/mL. Furthermore, 5-iodoindole had the same MICs against all seven clinical isolates of *A*. *baumannii* examined (Table S1). Of these strains A 550, A 578, and A 553 were effective biofilm producers, A 556, A 580, and A 571 were intermediate, and A 564 was poor [30]. 5-Iodoindole also dose-dependently inhibited biofilm formation by the two MDR strains, A 553 (sensitive to colistin and amikacin) (Figure 1B), and A 578 (sensitive to colistin, imipenem, and meropenem) (Figure 1C). These observations indicate that the similar antibiofilm activities of 5-iodoindole on *A*. *baumannii* were mainly due to its antimicrobial activity.

### 3.2. Effects of 5-Iodoindole on Biofilm Dispersal and Biofilm Formation

**Figure 2:**
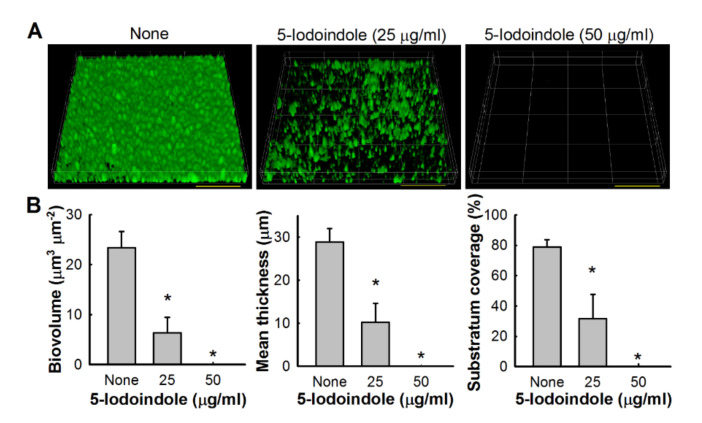
Biofilm inhibition of *A. baumannii* ATCC 17978 by 5-iodoindole. (**A**) Confocal Laser Scanning Microscopy observations of biofilm inhibition by 5-iodoindole. Scale bar = 50 µm; (**B**) Biofilm biomasses, mean thicknesses, and substratum coverages were quantified by COMSTAT analysis. Error bars represent standard deviation. * *p* < 0.05 versus untreated controls.

Bacterial biofilms are resistant to antibiotic treatment [42,43] at concentrations at least five times greater than their MICs [44]. Our biofilm dispersal assay results showed that 5-iodoindole could reduce preformed *A. baumannii* biofilms. For example, 5-iodoindole at 250 µg/mL reduced ATCC 17978 preformed biofilm by 62 ± 8% (Figure 1D), and living cell counts in biofilms showed nearly 95% of cells were killed by 5-iodoindole at 250 µg/mL. Similar results were obtained for the two clinical MDR isolates (A 553 and A 578) (Figure 1E,F). The antibiofilm effects of 5-iodoindole were further confirmed by confocal microscopy. *A. baumannii* ATCC 17978 readily formed biofilms, but the presence of 5-iodoindole at 25 µg/mL markedly reduced biofilm formation and at 50 µg/mL completely abolished biofilm formation (Figure 2A and Figure S3). Biofilm reductions were confirmed by COMSTAT analysis, which showed 5-iodoindole at 25 or 50 µg/mL significantly reduced biofilm biomass, thickness, and substrate coverage (Figure 2B).

### 3.3. Effects of 5-Iodoindole on Pellicle Formation and Surface Motility

**Figure 3:**
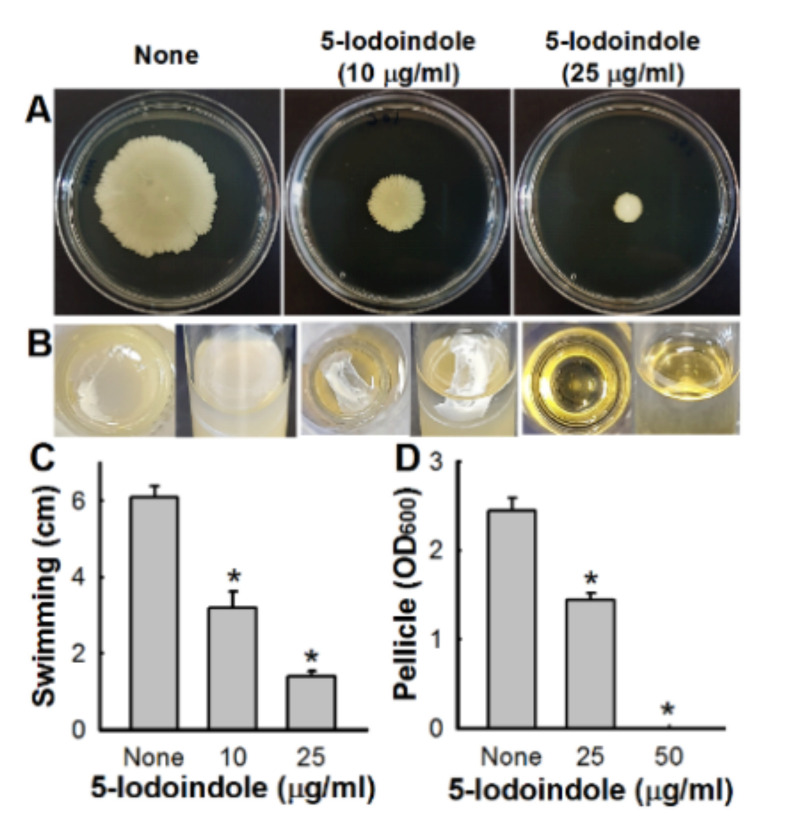
Effects of 5-iodoindole on motility and pellicle formation. (**A**) Surface motilities on 0.4% agarose were investigated after adding 5-iodoindole; (**B**) Pellicle inhibition by 5-iodoindole was studied by growing *A. baumannii* ATCC 17978 strain in the presence or absence of 5-iodoindole for 72 h at 25 °C; (**C**,**D**) The bar graph represents surface motility diameters (cm) and pellicle formation in the presence or absence of 5-iodoindole. Error bars represent standard deviation. * *p* < 0.05 versus untreated controls.

The motility of *A. baumannii* ATCC 17978 on 0.4% agar plates was significantly inhibited by 5-iodoindole at 10 or 25 µg/mL (Figure 3A,C). Since *A. baumannii* forms biofilms at air-liquid interfaces by forming pellicles [45], we investigated the effect of 5-iodoindole on ATCC 17978 pellicle formation after three days at 25 °C. As observed previously [30,46], nontreated *A. baumannii* formed robust pellicles by the end of the third day, and this was significantly inhibited by 5-iodoindole at 25 µg/mL and completely inhibited at 50 µg/mL (Figure 3B,D).

### 3.4. 5-Iodoindole Rapidly Induced A. baumannii Cell Death

**Figure 4:**
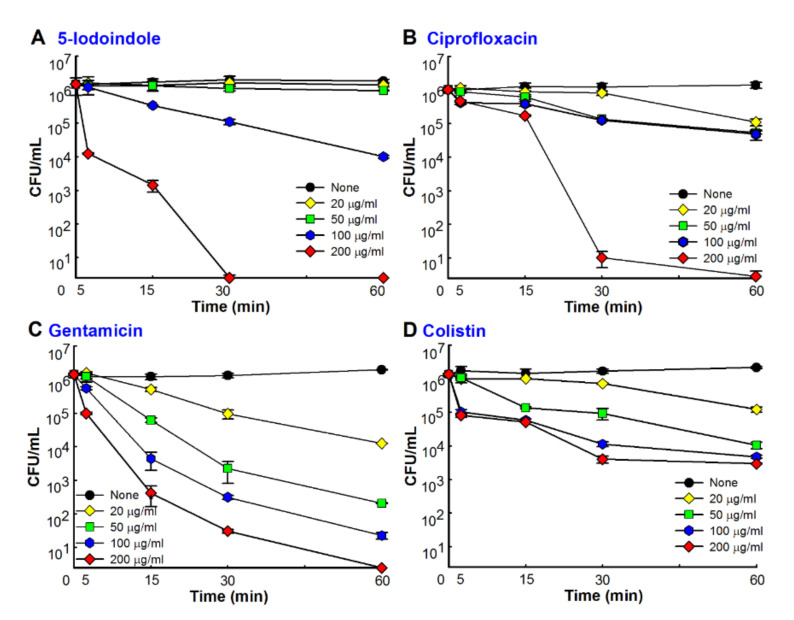
Rapid killing of *A. baumannii* by antibiotics and 5-iodoindole treated with 0, 20, 50, 100 or 200 µg/mL of (**A**) 5-iodoindole; (**B**) or ciprofloxacin; (**C**) or gentamycin; (**D**) or colistin. After incubation with antibiotics, cultures were plated, and colony forming units (CFUs) were enumerated. Error bars represent standard deviation.

Time-kill assays showed 5-iodoindole at 50 µg/mL required 24 h to achieve a 3-log reduction in cell viability and at 200 µg/mL rapidly killed *A. baumannii* with >3-log viable cell reduction within 15 min (Figure 4A). Similar rapid kinetics were observed for the commercial antibiotics ciprofloxacin, colistin, and gentamicin. Notably, 5-iodoindole killed *A. baumannii* as effectively as ciprofloxacin (Figure 4B) and better than gentamicin or colistin at same concentration (200 µg/mL) within one hour of exposure (Figure 4C,D). To investigate how 5-iodoindole kills *A. baumannii* cells, three other iodo compounds (sodium iodide, potassium iodide, and povidone iodine) and 15 other halogenated indoles were tested. However, the three iodo compounds at concentrations up to 500 µg/mL failed to kill *A. baumannii* cells after 24 h of incubation (data not shown). Of the 16 halogenated indoles, 5-, 6-, 7-, and 4-iodoindole (presented in decreasing order of activity) rapidly killed *A. baumannii* (Figure S1). These results suggest that the presence of the iodide ion is not a major factor, but rather that the position of the iodine on the indole skeleton determines bactericidal activity.

### 3.5. 5-Iodoindole Killing of A. baumannii Was Mediated by Cell Membrane Damage

**Figure 5:**
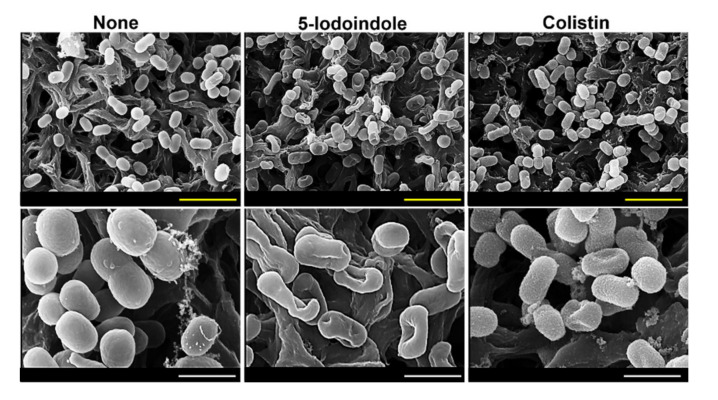
Membrane damage by 5-iodoindole in *A. baumannii* cells. Cells were grown with 5-iodoindole (125 µg/mL) or colistin (200 µg/mL; positive control) for 1 h and fixed on nylon membrane filters (0.45 µm). Scale bar: yellow = 3 µm, white = 1 µm.

To understand the mechanism responsible for 5-iodoindole induced cell death, we first investigated its effects on membrane integrity using colistin as a positive control, which has been reported to induce surface roughness and disrupt membrane integrity [39]. SEM analysis showed obvious cell shrinkage after treatment with 5-iodoindole at 125 µg/mL for 60 min (Figure 5 and Figure S4). Both 5-iodoindole and colistin caused visible recessed parts of membrane, though interestingly, 5-iodoindole induced smooth membrane surfaces while colistin caused surface roughening, which was in-line with the observed rapid and tardy bactericidal effects of 5-iodoindole and colistin (Figure 4A,D).

### 3.6. Effects of 5-Iodoindole on ROS Production and Release of Reducing Sugars and Proteins

**Figure 6:**
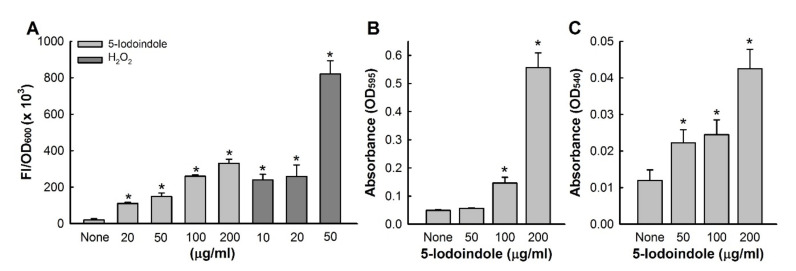
5-Iodoindole induced ROS production in and membrane release by *A. baumannii*. (**A**) Intracellular ROS production in *A. baumannii* cells treated with 5-iodoindole or H_2_O_2_; Comparative quantitative display of release of (**B**) proteins; (**C**) sugars after 3 h of treatment with 5-iodoindole. Error bars represent standard deviation. * *p* < 0.05 versus untreated controls.

Since it is well known that bacterial membrane disruption is caused by ROS generation [47], we investigated the effect of 5-iodoindole on intracellular ROS production. Treatment with 5-iodoindole significantly and dose-dependently increased ROS levels (Figure 6A). For example, 5-iodoindole at 20 or 200 µg/mL increased ROS production 6- and 17-fold, respectively, whereas the H_2_O_2_ (the positive control) at 20 µg/mL increased ROS production 13-fold. We also examined the effects of 5-iodoindole for 3 h on the membrane release of reducing sugars and proteins. As was expected, 5-iodoindole caused significant and dose-dependent losses of cytoplasmic proteins and sugars (Figure 6B,C). For example, protein release was 11.2-fold higher after treatment with 5-iodoindole at 200 µg/mL than for nontreated controls (Figure 6B). Similarly, sugar release was 3.5-fold higher after treatment with 5-iodoindole at 200 µg/mL than for nontreated controls (Figure 6C).

### 3.7. Antimicrobial Activities of 5-Iodoindole against Other Bacteria and the Fungus *C. albicans*

**Figure 7:**
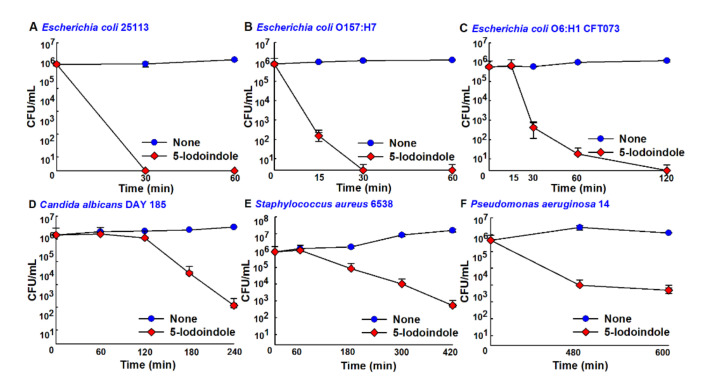
Rapid killing of other microbes by 5-iodoindole. (**A**) *E. coli* BW25113; (**B**) *E. coli* O157:H7; (**C**) *E. coli* O6:H1 CFT073; (**D**) *C. albicans* DAY185; (**E**) *S. aureus* ATCC 6538; (**F**) *P. aeruginosa* 14 cultures were treated with 5-iodoindole at 486 µg/mL. At the indicated time points, aliquots of treated cells were plated and CFUs were enumerated. Error bars represent standard deviation.

**Figure 8:**
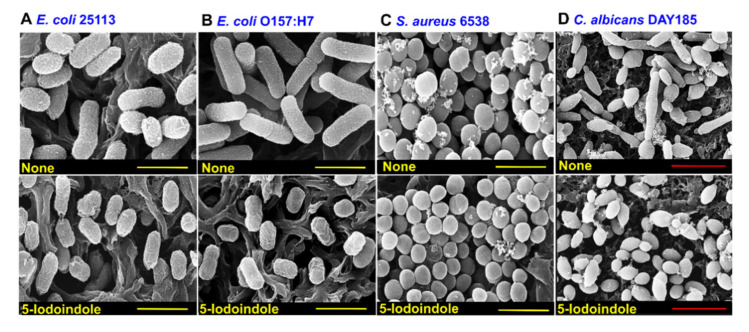
Morphological change by 5-iodoinole in other microbial cells. (**A**) *E. coli* BW25113; (**B**) *E. coli* O157:H7; (**C**) *S. aureus* ATCC 6538; (**D**) *C. albicans* DAY185 cells were grown with or without 5-iodoindole at 484 µg/mL for 2 h and then fixed on nylon membrane filters. Scale bars: yellow = 1 µm, red = 10 µm.

The effects of 5-iodoindole was investigated against three *E. coli* strains (commensal *E. coli* K-12 BW25113, enterohemorrhagic *E. coli* O157:H7, and uropathogenic *E. coli* O6:H1), Gram-positive *S. aureus*, Gram-negative *P. aeruginosa*, and the fungus *C. albicans*. Notably, 5-iodoindole at 486 µg/mL killed all three *E. coli* strains within 2 h (Figure 7A–C), *C. albicans* within 4 h (Figure 7D), and *S. aureus* within 7 h (Figure 7E), while the killing efficacy was less appreciable against *P. aeruginosa* (Figure 7F), which is in-line with our previous findings [22]. In accord with their observed killing effects, SEM analysis showed 5-iodoindole induced cell shrinkage and loss of cell membrane or wall in *E. coli* BW25113 and *E. coli* O157:H7 (Figure 8). Interestingly, treatment with 5-iodoindole decreased cell length in two *E. coli* strains (*E. coli* BW25113 and *E. coli* O157:H7 EDL933) and *C. albicans* but did not change the cell morphology and cell size of *S. aureus*. These results suggest that rapid killing of *A. baumannii* and *E. coli* strains by 5-iodoindole might be due to loss of cell wall/membrane integrity.

## 4. Discussion

Increasing evidence indicates that indole and its derivatives have antimicrobial and antibiofilm activities against multi-drug resistant bacteria [12,18,22]. Halogenated arenes such as 4-chloroindole-3-acetic acid, iodolactone, 3-chlorotyrosine, 3-bromotyrosine, and 5-bromouracil [48,49] are important in the pharmaceutical, agricultural, chemical, and material sciences [49]. Of the 24 indole derivatives examined, four potently inhibited *A. baumannii* biofilm formation in vitro (Table S3). Furthermore, 5-iodoindole effectively inhibited biofilm formation and growth and promoted *A*. *baumannii* biofilm dispersal (Figure 1). Notably, 5-iodoindole was found to more quickly kill *A. baumannii* than the other antibiotics examined (ciprofloxacin, colistin, and gentamicin) (Figure 4).

The majority of antibiotics and antimicrobial peptides that lyse bacterial membranes are fast acting and display detectable lysis within 15 to 90 min [5,50]. We found 5-iodoindole acted quickly and dose-dependently. While speculative, 5-iodoindole has multiple targets in addition to the wall/membrane damage (Figure 5) and ROS production (Figure 6). Other bactericidal disinfectants like chlorhexidine have been reported to increase ROS production and to damage various cellular macromolecules, such as RNA, DNA, protein, and lipids, in *A. baumannii*, and thus, cause cell death [51]. Indole derivatives such as indole-3-carbinol and its dimeric product, 3,3′-diindolylmethane have been reported to be effective against a number of human cancers in vitro and in vivo and to induce cell death via various pathways, which include interruptions of DNA and/or RNA synthesis and the inhibition of cell division [52]. In addition, bisindole has been reported to target DNA synthesis and provide broad spectrum antimicrobial activity [53].

Membrane permeability is typically limited when the polar surface area (PSA) of a molecular entity exceeds 140 Å^2^ [54], and thus, with a PSA of 15.8 Å^2^, 5-iodoindole would appears to be membrane permeable. The efficacies of indole derivatives such as 2, 5, 6- and 2, 5, 7-trisubstituted benzimidazoles in *M. tuberculosis*, *S. aureus*, and *B. subtilis* are reflected by their abilities to target a key functional protein in bacterial cell division like FtsZ. In the same study, it was hypothesized that these derivatives bound in the interdomain cleft of FtsZ, interrupted GTPase activity, stimulated FtsZ polymerization, and ultimately inhibiting bacterial cell division [55]. Since 5-iodoindole decreased cell size of *E. coli* strains and *C. albicans* (Figure 8) and was found to rapidly kill *A. baumannii* (Figure 4), other microbes (Figure 7), three nematode species [24,25,26,27], and an insect [25], but not plants or vertebrates [25,26], it would appear that 5-iodoindole is selectively transported through the membranes of bacteria, fungi, and nematodes and then specifically targets DNA.

Regarding the chemical structures of halogenated indoles, the additions of halogen atoms (fluorine, chlorine, bromine, or iodine) to the indole moiety have dissimilar antibacterial and antibiofilm effects (Tables S2 and S3). As elemental halogens are often toxic and highly reactivity while iodine is the least reactive of the four common halogens. The electronegativities of the halogen decrease with atomic weight as do bond lengths and carbon to halogen bond strengths. Hence, C–I bond is easiest to break to form free radicals compared to C–F, C–Br, and C–Cl bond. According to electron withdrawing resonance studies [56], the C_5_ and C_7_ carbon positions of indoles are electron rich and by considering properties of C–I bond and resonance effect at C_5_ position in 5-iodoindole makes it most effective and rapid antimicrobial agent against *A. baumannii*. Interestingly, 6-iodoindole exhibited antibiofilm activity similar to 5-iodoindole and >2-log reduction in cell growth, whereas 7-iodoindole did not have any significant effect (Figure S1). Additionally, our in vitro studies showed other lighter halogen containing indole derivatives such as 4-fluroindole, 5-fluroindole, 6-fluroindole, and 7-fluroindole have not shown effective anti-*baumannii* activity. Of the halogenated indoles tested, 5-iodoindole and 6-iodoindole most effectively controlled *A. baumannii* cell growth and biofilm formation (Figure S1). Previously, 5-iodoindole was reported to be nontoxic in a mouse model and in plant germination models [24,25,26], but to rapidly kill pine wood [25] and root-knot nematodes [26], which in combination with our findings suggests 5-iodoindole is relatively safe for biological applications.

## 5. Conclusions

This is the first report to suggest 5-iodoindole be considered a control agent for MDR *A. baumannii* and other microbes. Since hundreds of indole derivatives are commercially accessible and new indole derivatives are being synthesized [22,57], we suggest screening of larger libraries of indole derivatives be performed to identify agents capable of eradicating human pathogens like pathogenic *A. baumannii*. In particular, given the growing incidence of MDR *A. baumannii* strains, novel therapeutics are required to address this pathogen. The present study provides details of the mode action of 5-iodoindole and offers hints that might aid the comprehensive elucidation of the mechanism involved. Importantly, our findings suggest iodine–indole compounds might serve as prototypes for the developments of novel anti-*baumannii* drugs.

## Supplementary Materials

The following are available online at https://www.mdpi.com/2218-273X/10/8/1186/s1, Figure S1: Rapid killing of *A. baumannii* ATCC 17978 by halogenated indoles, Figure S2: Biofilm inhibition of *A. baumannii* by 6-iodoindole, Figure S3: Additional confocal microscopic images showing biofilm inhibition of *A. baumannii*, Figure S4: Additional SEM images showing membrane damage by 5-iodoindole in *A. baumannii* cells Table S1: Determination of minimum inhibitory concentrations (MICs) of 5-iodoindole for *A. baumannii* ATCC 17978 and clinical isolates. MIC determinations after culture for 24 h at 37°C in 96-well plates in the presence of 5-iodoindole, Table S2: MICs of halogenated indole derivatives against *A. baumannii* ATCC 17978, Table S3: Antibiofilm and antibacterial activities of indole compounds against *A. baumannii* ATCC 17978.
