# Supplementary material for: Rapid Killing and Biofilm Inhibition of Multidrug-Resistant Acinetobacter baumannii Strains and Other Microbes by Iodoindoles
Source: Biomolecules. 2020 Aug 14;10(8):1186. doi: 10.3390/biom10081186 (PMC7465641; doi:10.3390/biom10081186)
Supplement: Supplementary file 1 [file biomolecules-10-01186-s001.pdf]

biomolecules

Rapid Killing and Biofilm Inhibition of Multidrug-Resistant *Acinetobacter baumannii* Strains and Other Microbes by Iodoindoles

Chaitany Jayprakash Raorane †, Jin-Hyung Lee † and Jintae Lee *

School of Chemical Engineering, Yeungnam University, Gyeongsan 38541, Republic of Korea; chaitanyaraorane22@ynu.ac.kr (C.J.R.); jinhlee@ynu.ac.kr (J.-H.L.)

* Correspondence: jtlee@ynu.ac.kr; Tel.: +82-53-810-2533; Fax: +82-53-810-4631

† These authors contributed equally to this work.

Supplementary data

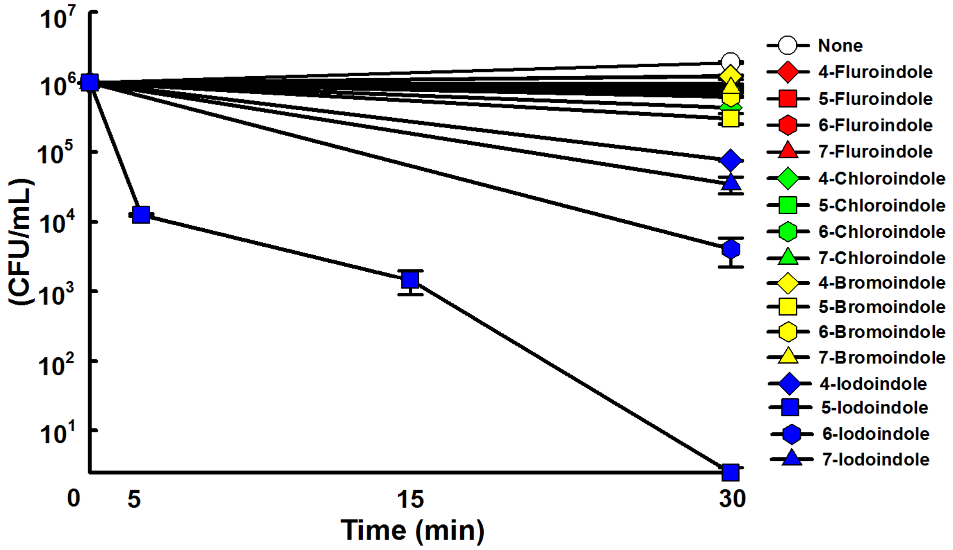

Figure S1. Rapid killing of *Acinetobacter baumannii* ATCC 17978 by halogenated indoles. *A. baumannii* cultures were treated with 0 or 200 µg/ml of the listed halogenated indoles. After incubation, cultures were plated, and CFUs were enumerated. Error bars represent standard deviation.

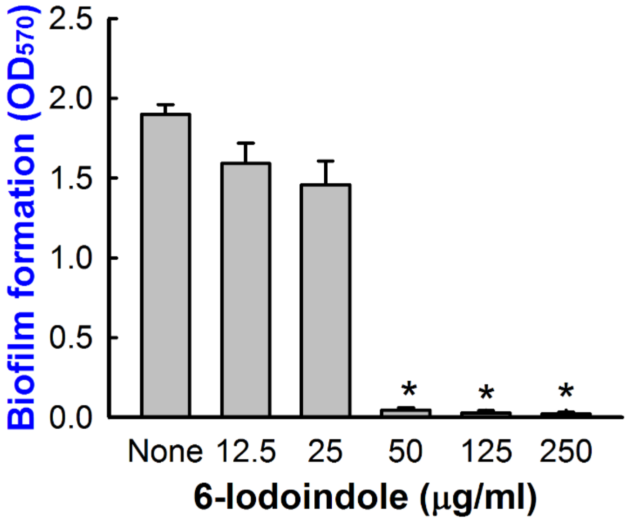

Figure S2. Biofilm inhibition of *A. baumannii* by 6-iodoindole. Biofilm inhibitory effects of 6-iodoindole against *A. baumannii* strains ATCC 17978 after culture for 24 h at 37°C without shaking. Error bars represent standard deviation. *$p < 0.05$ versus untreated controls.

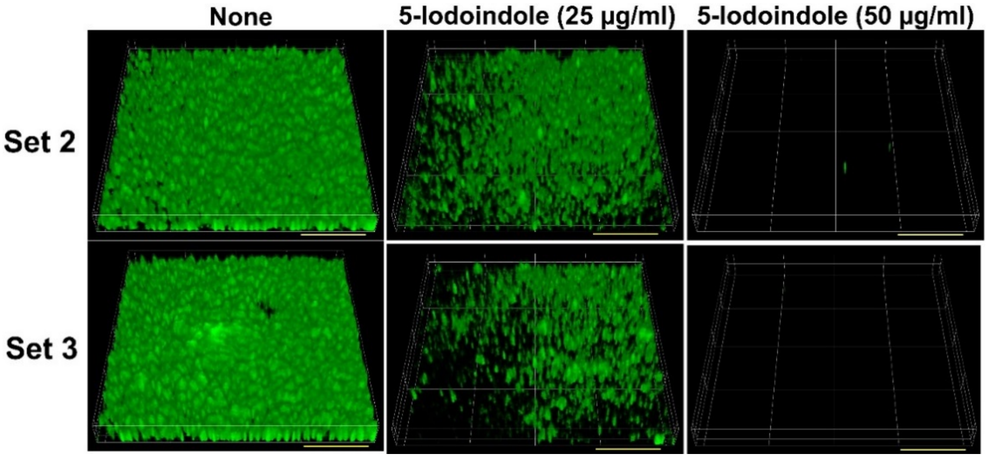

Figure S3. Additional confocal microscopic images showing biofilm inhibition of *A. baumannii*. Scale bar = 50 μm.

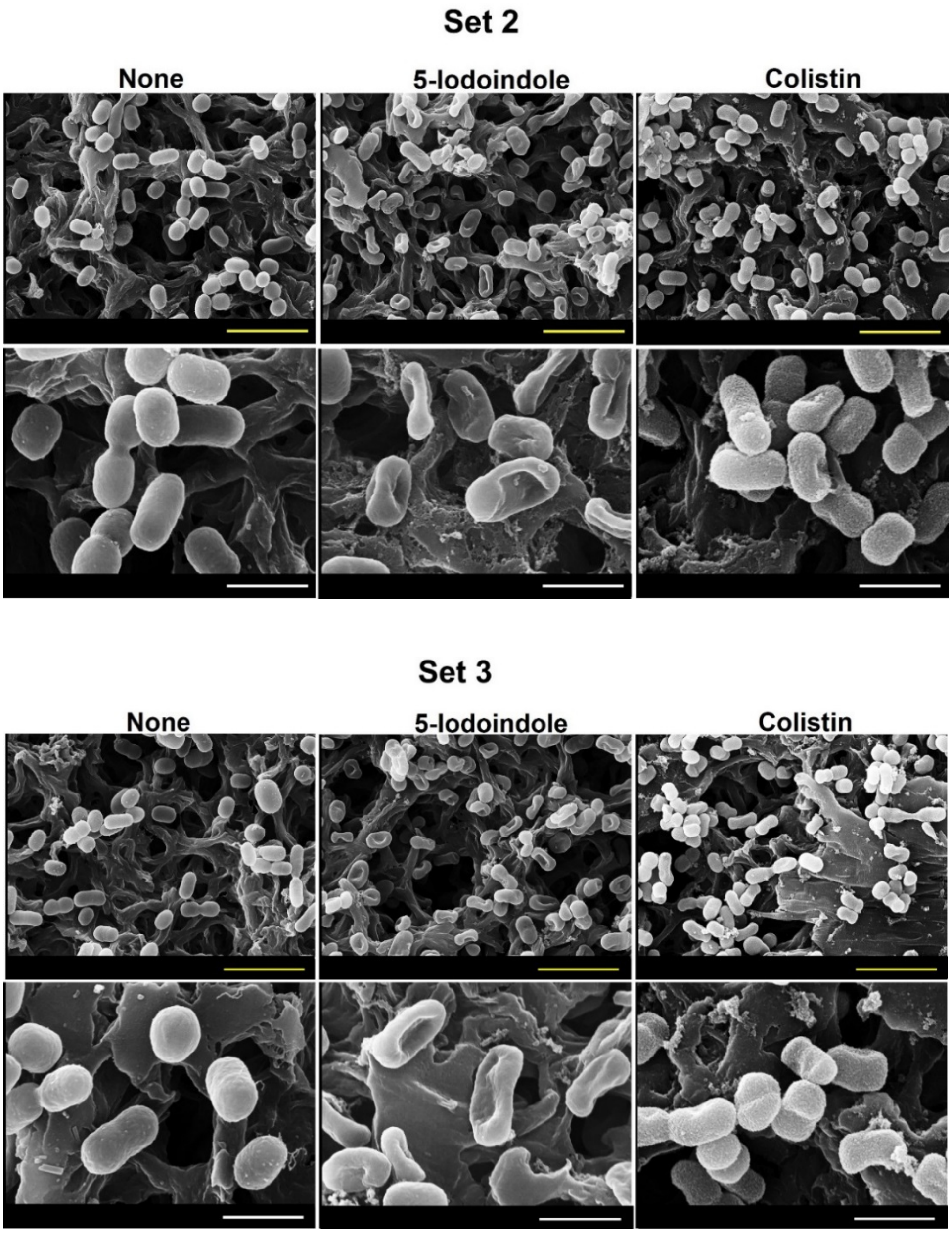

Figure S4. Additional SEM images showing membrane damage by 5-iodoindole in *A. baumannii* cells. Cells were grown with or without 5-iodoindole (125 µg/ml) and or colistin (200 µg/ml; positive control) for 1 h and fixed on nylon membrane filters (0.45 µm). Scale bar: yellow = 3 µm, white = 1 µm.

Table S1. Determination of minimum inhibitory concentrations (MICs) of 5-iodoindole for *A. baumannii* ATCC 17978 and clinical isolates. MIC determinations after culture for 24 h at 37°C in 96-well plates in the presence of 5-iodoindole.

	A. baumannii **strain**	**5-Iodoindole MIC (µg/ml)**	**Antibiotic**
**Reference strain**	ATCC 17978	50	Sensitive
	ATCC BAA-1709	50	Sensitive
**Clinical isolates obtained from burns patients at the National Rehabilitation Institute of Mexico**	A 550	50	MDR
	A 553	50	MDR
	A 556	50	MDR
	A 564	50	MDR
	A 571	50	Sensitive
	A 578	50	MDR
	A 580	50	MDR

Table S2. MICs of halogenated indole derivatives against *A. baumannii* ATCC 17978.

Chemical name	MIC (µg/ml)
4-Bromoindole	125
5-Bromoindole	125
6-Bromoindole	125
7-Bromoindole	125
4-Chloroindole	100
5-Chloroindole	125
6-Chloroindole	125
7-Chloroindole	200
4-Fluroindole	125
5-Fluroindole	125
6-Fluroindole	250
7-Fluroindole	>250
4-Iodoindole	100
5-Iodoindole	50
6-Iodoindole	50
7-Iodoindole	125

Table S3. Antibiofilm and antibacterial activities of indole compounds against *A. baumannii* ATCC 17978. The biofilm screening of *A. baumannii* ATCC 17978 was performed after culture in TSB for 24 h at 37°C in 96-well polystyrene plates in the presence of indole derivatives at 50 µg/ml.

Name	Structure	Biofilm (%)	Cell growth (%)
**None**		100	100
**4-Bromoindole**		38 ± 8	32 ± 7
**5-Bromoindole**		90± 7	66 ± 3
**6- Bromoindole**		114 ± 8	68 ± 7
**7- Bromoindole**		106 ± 9	93 ± 1
**5-Bromo-3-iodo-7-azaindole**		84 ± 9	96 ± 4
**4-Chloroindole**		25 ± 9	28 ± 5
**5-Chloroindole**		119 ± 17	50 ± 9
**6-Chloroindole**		90 ± 5	70 ± 10
**7-Chloroindole**		98 ± 9	88 ± 2
**5,6-Difluro isatin**		112 ± 3	92 ± 2
**2,3-Dimethyl indole**		54 ± 12	80 ± 10
**4-Fluroindole**		109 ± 3	89 ± 2

Name	Structure	Biofilm (%)	Cell growth (%)
**5-Fluroindole**		121 ± 14	87 ± 7
**6-Fluroindole**		118 ± 5	98 ± 2
**7-Fluroindole**		127 ± 8	91 ± 2
**7-Fluoro-5-iodoindole-3-carboxaldehyde**		75 ± 4	102 ± 4
**Indole**		93 ± 7	100 ± 1
**4-Iodoindole**		40 ± 10	90 ± 11
**5-Iodoindole**		**4 ± 1**	**2 ± 1**
**6-Iodoindole**		**6 ± 1**	**5 ± 2**
**7-Iodoindole**		94 ± 8	79 ± 5
**Isatin**		75 ± 6	94 ± 2
**3-Methyl indole**		114 ± 7	81 ± 2
**7-Nitroindole**		87 ± 10	59 ± 5
**5-Nitroisatin**		82 ± 3	102 ± 1
**5-(Trifluro methoxy) indoline -2,3-dione**		92 ± 2	101 ± 3

Table S3. continued
